# Chemotherapy for primary mediastinal yolk sac tumor in a patient undergoing chronic hemodialysis: a case report

**DOI:** 10.1186/s13256-017-1213-7

**Published:** 2017-02-16

**Authors:** Haruki Hirakawa, Chiho Nakashima, Tomomi Nakamura, Masanori Masuda, Taro Funakoshi, Shunsaku Nakagawa, Takahiro Horimatsu, Kazuo Matsubara, Manabu Muto, Shinya Kimura, Naoko Sueoka-Aragane

**Affiliations:** 1 Division of Hematology, Respiratory Medicine and Oncology, Department of Internal Medicine, Faculty of Medicine, Naoko Sueoka-Aragane, Saga University, 5-1-1 Nabeshima, Saga, 849-8501 Japan; 2 Department of Pathology, Faculty of Medicine, Saga University Hospital, Saga, Japan; 3 Department of Therapeutic Oncology, Graduate School of Medicine, Kyoto University, Kyoto, Japan; 4 Department of Pharmacy, Kyoto University Hospital, Kyoto, Japan

**Keywords:** Mediastinal yolk sac tumor, Hemodialysis, Renal insufficiency, Cisplatin, Pharmacokinetics

## Abstract

**Background:**

The safety and efficacy of chemotherapy for patients undergoing concomitant hemodialysis have not been fully established and optimal doses of anti-cancer drugs and best timing of hemodialysis remains unclear. Although chemosensitive cancers, such as germ cell tumors, treated with chemotherapy should have sufficient dose intensity maintained to achieve the desired effect, many patients with cancer undergoing hemodialysis might be under-treated because the pharmacokinetics of anti-cancer drugs in such patients remains unknown.

**Case presentation:**

We describe a 31-year-old Japanese man with a mediastinal yolk sac tumor treated with surgery followed by five cycles of chemotherapy containing cisplatin and etoposide while concomitantly undergoing hemodialysis. The doses of these agents used in the first cycle were 50% of the standard dose of cisplatin (10 mg/m^2^) and 60% of the standard dose of etoposide (60 mg/m^2^) on days 1 through to 5; the doses were subsequently escalated to 75% with both agents. Hemodialysis was started 1 hour after infusions of these agents. Severe hematological toxicities were observed despite successful treatment. During treatment with concurrent hemodialysis, pharmacokinetic analysis of cisplatin was performed and its relationship with adverse effects was assessed. Compared with patients with normal renal function, the maximum drug concentration was higher, and concentration increased in the interval between hemodialysis and the subsequent cisplatin infusion, resulting in a higher area under the curve despite a reduction in the dose to 75% of the standard regimen.

**Conclusions:**

Because of the altered pharmacokinetics pharmacodynamics status of patients with renal dysfunction undergoing hemodialysis, pharmacokinetics pharmacodynamics analysis is deemed to be helpful for effective and safe management of chemotherapy in patients undergoing hemodialysis.

## Background

Germ cell tumors of extragonadal origin have been reported to make up from 1 to 5% of all germ cell tumors. The most common site is the mediastinum, constituting 50 to 70% [1, 2]. In general, germ cell tumors are sensitive to platinum-based chemotherapy and patients have a favorable prognosis. However, response of mediastinal non-seminomatous germ cell tumors to therapy is less than favorable, so these tumors have been classified into the poor prognosis group [3, 4]. It has been reported that 40 to 54% of patients with mediastinal non-seminomatous germ cell tumor treated with platinum-based chemotherapy followed by surgical resection achieved long-term disease-free survival, but patients who relapsed after initial treatment experienced dismal outcomes with only 10% long-term survival [5–7]. Therefore, it is important to accomplish successful initial chemotherapy with an adequate dose.

Recently, the number of patients with end-stage renal failure undergoing hemodialysis (HD) is increasing, and patients undergoing HD are potentially at high risk of cancer [8]. However, the safety and efficacy of chemotherapy for patients undergoing concomitant HD have not been fully established and optimal doses of anti-cancer drugs and best timing of HD remains unclear. Many patients with cancer undergoing HD might be under-treated because the pharmacokinetics (PK) of anti-cancer drugs in such patients remains unknown and severe adverse effects are feared. However, chemosensitive cancers such as germ cell tumors treated with chemotherapy should have sufficient dose intensity maintained to achieve the desired effect.

In this article, we report the case of a patient with mediastinal yolk sac tumor who was successfully treated with cisplatin and etoposide while concomitantly undergoing HD. We carried out a PK analysis of cisplatin as part of a multicenter study and carefully considered the relationship between adverse effects and blood concentration of cisplatin.

## Case presentation

**Fig. 1:**
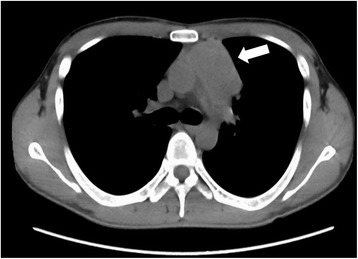
Computed tomography scan confirms a solid tumor (53×35 mm) in the anterior mediastinum (*arrow*)

**Fig. 2:**
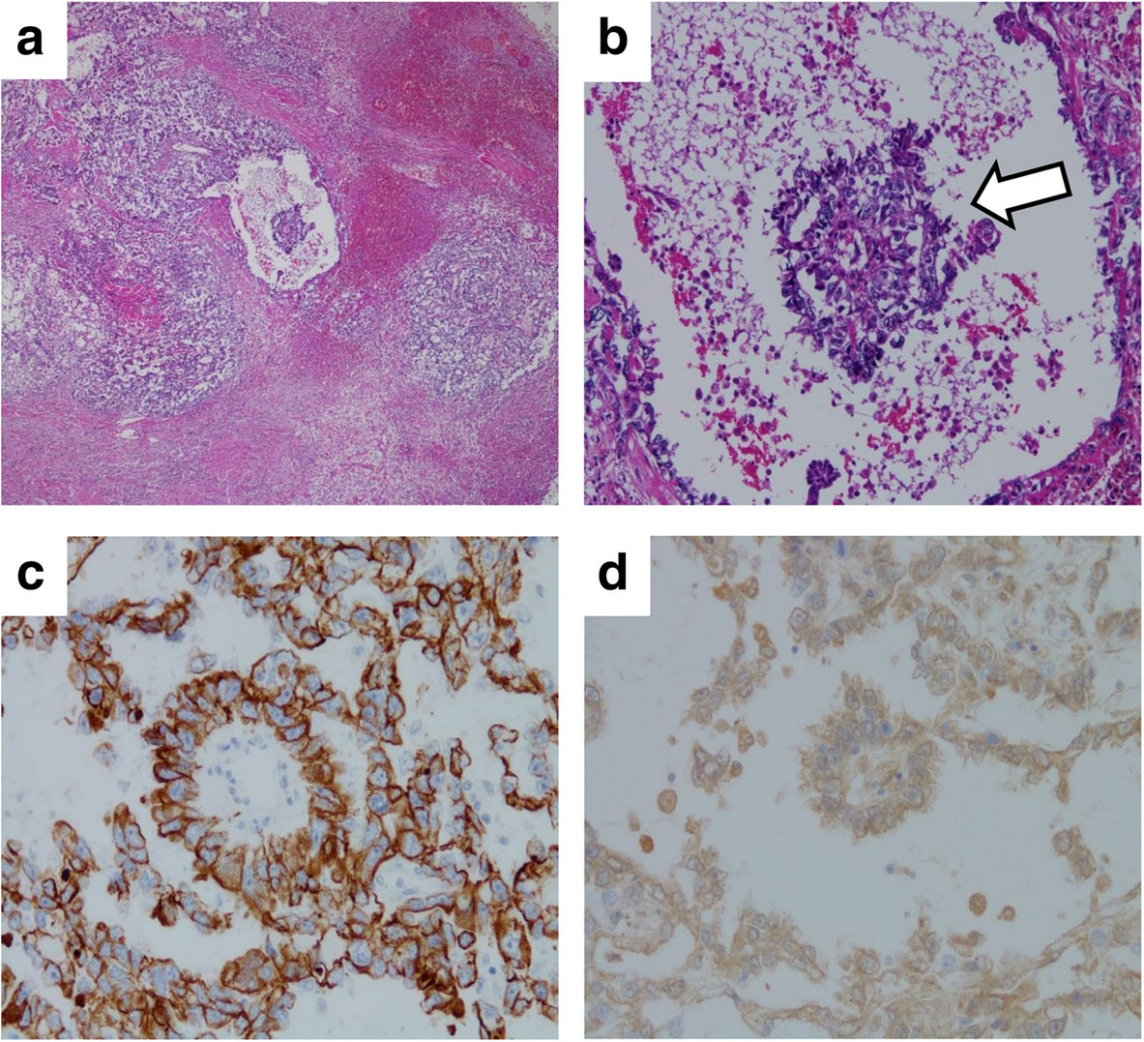
Pathological findings of computed tomography-guided biopsy specimen. **a** Tumor cells with mixed sinusoidal-like, cystic, and papillary structures were observed (hematoxylin and eosin staining, ×20). **b** Schiller–Duval body was highlighted with *white arrow* (hematoxylin and eosin staining, ×100). Immunohistochemical staining is positive for cytokeratin AE1/AE3 (×200) (**c**) and alpha-fetoprotein (**d**) (×200)

A 31-year-old Japanese man was referred to our hospital with renal dysfunction in April 2014. He had a history of hypertension, hyperuricemia, and pituitary dwarfism, but had not taken any medications. He was 159.4 cm in height and 42.6 kg in weight (body surface area 1.398 m^2^). Initial blood tests showed anemia and renal dysfunction: blood urea nitrogen (BUN) 64.6 mg/dl, creatinine (Cr) 5.66 mg/dl. A chest X-ray performed to evaluate cardiomegaly revealed a mediastinal tumor in his right thorax; a computed tomography (CT) scan confirmed the mass to be a well-defined, homogeneous solid tumor (53×35 mm) in his anterior mediastinum (Fig. 1). An additional blood examination showed a high level, 322.6 ng/ml, of alpha-fetoprotein (AFP, normal <9 ng/ml), but his beta-human chorionic gonadotropin (β-hCG) level was within normal range. A CT-guided biopsy of the mediastinal tumor showed the presence of tumor cells with mixed sinusoidal-like, cystic, and papillary structures (Fig. 2a) and Schiller–Duval body (Fig. 2b), which was positive by immunohistochemistry (IHC) for cytokeratin (CK) AE1/AE3 and AFP, and negative for placental alkaline phosphatase (PLAP), β-hCG, and CD30 (Fig. 2c, d), resulting in a diagnosis of primary mediastinal yolk sac tumor. Because of the complication of end-stage renal insufficiency, surgery was selected as the first treatment; following surgery, his AFP level decreased to the normal range. Subsequently, chemotherapy with cisplatin (10 mg/m^2^) and etoposide (60 mg/m^2^) was conducted daily from day 1 through to day 5. HD was started 1 hour after infusions of these agents. Appendicitis with grade 4 neutropenia occurred, so an appendectomy was performed after the first cycle. In addition, grade 3 anemia and grade 4 neutropenia appeared under prophylactic treatment with granulocyte-colony stimulating factor (G-CSF). Because severe hematological toxicities occurred, we conducted chemotherapy without dose escalation on the second cycle. In contrast to the first cycle, toxicities were admissible with 10 mg/m^2^ cisplatin and 60 mg/m^2^ etoposide in the second cycle. To elevate the dose intensity, the doses were escalated from the third to the fifth cycles: cisplatin to 15 mg/m^2^ and etoposide to 75 mg/m^2^. Grade 4 neutropenia and thrombocytopenia as well as grade 3 anemia were sustained over 7 days in spite of prophylactic treatment with G-CSF. After five cycles of chemotherapy, his AFP level remained in the normal range and there has been no recurrence for 1 year.

**Fig. 3:**
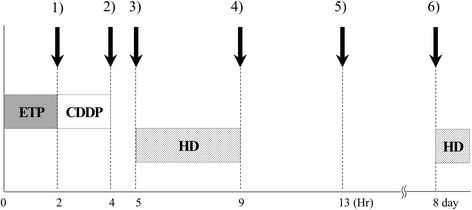
Schedule of chemotherapy, hemodialysis, and blood sampling. In the third and fourth chemotherapy cycles, cisplatin (15 mg/m^2^) and etoposide (75 mg/m^2^) was conducted daily from day 1 through to day 5. Hemodialysis was started 1 hour after infusions of these agents. Venous blood samples were collected five times each day: (*1*) before cisplatin infusion, (*2*) immediately after infusion, (*3*) before hemodialysis, (*4*) after hemodialysis, and (*5*) 4 hours after hemodialysis on days 1 to 5. In addition, blood was collected (*6*) before hemodialysis on day 8 once in each course. *CDDP* cisplatin, *ETP* etoposide, *HD* hemodialysis

**Fig. 4:**
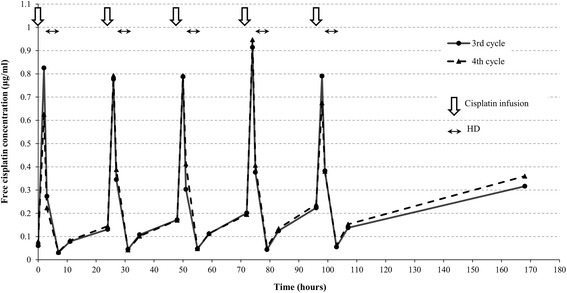
Free cisplatin concentration analysis. The *solid* and *dotted lines* show plasma free cisplatin concentration in the third and fourth chemotherapy cycles, respectively. *White arrows* indicate cisplatin (15 mg/m^2^) infusions and *black two-headed arrows* indicate hemodialysis during days 1 to 5. *HD* hemodialysis

During the third and fourth cycles, free cisplatin blood concentrations were measured as part of a multicenter study. Venous blood samples were collected five times each day: (1) before cisplatin infusion, (2) immediately after infusion, (3) before HD, (4) after HD, and (5) 4 hours after HD on days 1 to 5. In addition, blood was collected before HD on day 8 once in each course (Fig. 3). The time-concentration curve in the third cycle is shown in Fig. 4. Our patient was administered 15 mg/m^2^ cisplatin, and maximum concentration (Cmax) of free cisplatin was 0.8 to 0.9 μg/mL. Just before treatment with cisplatin, the concentration had not recovered to the level observed after the previous HD. The concentration before infusion of cisplatin gradually increased each day during the 5 days of treatment, and this phenomenon was observed until 8 days after the start of chemotherapy. During the fourth cycle, chemotherapy was administered using the same doses used in the third cycle, and PK was similar to that of the third cycle.

## Discussion

In the International Germ Cell Consensus Classification (IGCCC), primary mediastinal yolk sac tumor is classified into the poor prognosis group, and standard treatment consists of induction chemotherapy such as BEP (bleomycin, etoposide, and cisplatin) or VIP (ifosfamide, etoposide, and cisplatin) regimens followed by radical operation [4]. In our case, it was considered likely that standard chemotherapy would not be efficacious because of end-stage renal insufficiency, so surgery was selected as the first treatment so as not to miss the opportunity for complete tumor resection. In addition, bleomycin and active metabolite of ifosfamide are known not to be sufficiently removed by HD, resulting in enhanced toxicities such as pulmonary fibrosis, disturbance of consciousness, and convulsions [9–14]. Therefore, we selected combination chemotherapy with cisplatin and etoposide, and doses of these agents used in the first cycle were 50% of the standard dose of cisplatin (10 mg/m^2^) and 60% of the standard dose of etoposide (60 mg/m^2^) on days 1 through 5 according to previous reports, and the doses were subsequently escalated to 75% with both agents [14]. However, the rationale for using these dosages, especially that of cisplatin, has not been elucidated because of a paucity of PK studies involving patients with HD. Froehner *et al.* reported that they started the treatment from 50% reduction of cisplatin according to guidelines on testicular cancer, and then escalated to 100% of standard dose after monitoring adverse effects [14].

In this case, we obtained PK results for cisplatin during cycles 3 and 4 in cooperation with Kyoto University. The findings are as follows, compared with patients with normal renal function administered 100% standard doses of cisplatin [15]: (1) the Cmax of free cisplatin was higher, and (2) concentration gradually increased after HD up to the subsequent infusion of cisplatin (Fig. 4). The higher Cmax might be related to our patient’s low volume of distribution caused by pituitary dwarfism rather than renal insufficiency. Immediately after HD, the concentrations were lower, equivalent to those in patients with normal renal function based on previous reports [15]. However, his free cisplatin concentration rose 4 hours after HD, and gradual accumulation of free cisplatin was observed up until the subsequent treatment with cisplatin. Pronounced elevation of free cisplatin concentration may be due to redistribution from peripheral tissues and reduced excretion of free cisplatin, resulting in an elevated area under the curve (AUC) of free cisplatin leading to severe hematological toxicity [15–18]. Although nephrotoxicity has not been thought to be a limitation in HD patients, dose-related adverse effects are often observed [19], and dose reduction is inevitable just as in our case. Because of the altered PK pharmacodynamics (PD) status of patients with renal dysfunction undergoing HD, determination of cisplatin dosage should be based on monitored concentration to conduct effective and safe chemotherapy.

## Conclusions

Malignancies sensitive to chemotherapy require careful maintenance of dose intensity, but this should be performed safely. PK analysis is deemed to be useful for appropriate chemotherapy in patients undergoing HD. Additional evidence from other similar patients is needed to establish suitable chemotherapy regimens for patients with concomitant renal insufficiency.

## Abbreviations

AFP: Alpha-fetoprotein
AUC: Area under the curve
BEP: Bleomycin, etoposide, and cisplatin
β-hCG: Beta-human chorionic gonadotropin
BUN: Blood urea nitrogen
CK: Cytokeratin
Cmax: Maximum concentration
Cr: Creatinine
CT: Computed tomography
G-CSF: Granulocyte-colony stimulating factor
HD: Hemodialysis
IGCCC: International Germ Cell Consensus Classification
IHC: Immunohistochemistry
PD: Pharmacodynamics
PK: Pharmacokinetics
PLAP: Placental alkaline phosphatase
VIP: Ifosfamide, etoposide, and cisplatin

## References

[1] McKenney JK, Heerema-McKenney A, Rouse RV. Extragonadal germ cell tumors: a review with emphasis on pathologic features, clinical prognostic variables, and differential diagnostic considerations. Adv Anat Pathol. 2007;14:69–92. 10.1097/PAP.0b013e31803240e6 17471115

[2] Dehner LP. Germ cell tumors of the mediastinum. Germ cell tumors of the mediastinum. Semin Diagn Pathol. 1990;7:266–84. 2178277

[3] International Germ Cell Cancer Collaborative Group. International Germ Cell Consensus Classification: a prognostic factor-based staging system for metastatic germ cell cancers. J Clin Oncol. 1997;15:594–603. 10.1200/JCO.1997.15.2.594 9053482

[4] Schmoll HJ, Souchon R, Krege S, Albers P, Beyer J, Kollmannsberger C, European Germ Cell Cancer Consensus Group, *et al*. European consensus on diagnosis and treatment of germ cell cancer: a report of the European Germ Cell Cancer Consensus Group (EGCCCG). Ann Oncol. 2004;15:1377–99. 10.1093/annonc/mdh301 15319245

[5] Albany C, Einhorn LH. Extragonadal germ cell tumors: clinical presentation and management. Curr Opin Oncol. 2013;25:261–5. 10.1097/CCO.0b013e32835f085d 23422328

[6] Bokemeyer C, Nichols CR, Droz JP, Schmoll HJ, Horwich A, Gerl A, *et al*. Extragonadal germ cell tumors of the mediastinum and retroperitoneum: results from an international analysis. J Clin Oncol. 2002;20:1864–73. 10.1200/JCO.2002.07.062 11919246

[7] Rodney AJ, Tannir NM, Siefker-Radtke AO, Liu P, Walsh GL, Millikan RE, *et al*. Survival outcomes for men with mediastinal germ-cell tumors: the University of Texas M. D. Anderson Cancer Center experience. Urol Oncol. 2012;30:879–85. 10.1016/j.urolonc.2010.08.005 PMC3956468 20933444

[8] Maisonneuve P, Agodoa L, Gellert R, Stewart JH, Buccianti G, Lowenfels AB, *et al*. Cancer in patients on dialysis for end-stage renal disease: an international collaborative study. Lancet. 1999;354:93–9. 10.1016/s0140-6736(99)06154-1 10408483

[9] Superfin D, Iannucci AA, Davies AM. Commentary: Oncologic drugs in patients with organ dysfunction: a summary. Oncologist. 2007;12:1070–83. 10.1634/theoncologist.12-9-1070 17914077

[10] Eneman JD, Philips GK. Cancer management in patients with end-stage renal disease. Oncology (Williston Park). 2005;19:1199–214. 16255135

[11] Crooke ST, Luft F, Broughton A, Strong J, Casson K, Einhorn L. Bleomycin serum pharmacokinetics as determined by a radioimmunoassay and a microbiologic assay in a patient with compromised renal function. Cancer. 1977;39:1430–4. 10.1002/1097-0142(197704)39:4<1430::aid-cncr2820390412>3.0.co;2-v 66973

[12] Tomita M, Aoki Y, Tanaka K. Effect of haemodialysis on the pharmacokinetics of antineoplastic drugs. Clin Pharmacokinet. 2004;43:515–27. 10.2165/00003088-200443080-00002 15170366

[13] Carlson L, Goren MP, Bush DA, Griener JC, Quigley R, Tkaczewski I, *et al*. Toxicity, pharmacokinetics, and in vitro hemodialysis clearance of ifosfamide and metabolites in an anephric pediatric patient with Wilms’ tumor. Cancer Chemother Pharmacol. 1998;41:140–6. 10.1007/s002800050720 9443627

[14] Froehner M, Passauer J, Schuler U, Hakenberg OW, Wirth MP. Successful chemotherapy for advanced nonseminomatous germ-cell tumor in a patient undergoing chronic hemodialysis. J Clin Oncol. 2007;10:1282–4. 10.1200/JCO.2006.09.9549 17401020

[15] Ikeda K, Terashima M, Kawamura H, Takiyama I, Koeda K, Takagane A, *et al*. Pharmacokinetics of cisplatin in combined cisplatin and 5-fluorouracil therapy: a comparative study of three different schedules of cisplatin administration. Jpn J Clin Oncol. 1998;28:168–75. 10.1093/jjco/28.3.168 9614438

[16] Yamada Y, Ikuta Y, Nosaka K, Miyanari N, Hayashi N, Mitsuya H, *et al*. Successful treatment of Cisplatin overdose with plasma exchange. Case Rep Med. 2010;2010:802312. 10.1155/2010/802312 PMC2837905 20300587

[17] Chu G, Mantin R, Shen YM, Baskett G, Sussman H. Massive cisplatin overdose by accidental substitution for carboplatin. Toxic Manag Cancer. 1993;72:3707–14. 10.1002/1097-0142(19931215)72:12<3707::aid-cncr2820721224>3.0.co;2-u 8252487

[18] Schellens JH, Ma J, Planting AS, van der Burg ME, van Meerten E, de Boer-Dennert M, *et al*. Relationship between the exposure to cisplatin, DNA-adduct formation in leucocytes and tumour response in patients with solid tumours. Br J Cancer. 1996;73:1569–75. 10.1038/bjc.1996.296 PMC2074557 8664132

[19] Janus N, Thariat J, Boulanger H, Deray G, Launay-Vacher V. Proposal for dosage adjustment and timing of chemotherapy in hemodialyzed patients. Ann Oncol. 2010;21:1395–403. 10.1093/annonc/mdp598 20118214

